# Depth-resolved phase velocity estimation in layered tissue based on an efficient additive attention network with surface acoustic wave – optical coherence elastography

**DOI:** 10.1364/BOE.593027

**Published:** 2026-04-21

**Authors:** Guangyu Zhang, Jinpeng Liao, Zhengshuyi Feng, Katrien Van Bocxlaer, Alison M. Layton, Chunhui Li, Zhihong Huang

**Affiliations:** 1 Healthcare Engineering, School of Physics and Engineering Technology, University of York, UK; 2 Biomedical Engineering, School of Science and Engineering, University of Dundee, UK; 3 Hull York Medical School, University of York, York, UK

## Abstract

Optical coherence elastography (OCE) is a non-invasive imaging technique used to quantify tissue stiffness and to assist in the diagnosis and assessment of disease. A major limitation of conventional OCE approaches is that phase velocity estimation requires transformation from the spatial–temporal domain to the frequency–wavenumber domain, a process that is computationally inefficient and may introduce errors due to assumptions regarding tissue properties. We propose a unified framework for depth-resolved phase velocity estimation that combines spectral analysis of complex-valued signals with a deep learning inversion network. The effectiveness of the framework is validated using homogeneous agar phantoms, while layered agar phantoms and in vivo human skin are analyzed by depth-dependent phase velocity gradients. The proposed phase velocity estimation network (PVNet) achieved a mean absolute error (MAE) of 0.123 ± 0.024 m/s in agar models and 0.145 ± 0.114 m/s in human skin, compared with ground truth measurements. This study presents a deep learning approach for segmenting depth-resolved bi-layers in OCE, offering significant potential for the clinical identification of sub-surface lesions and abnormalities.

## 1. Introduction

The skin is the largest organ of the human body, acting as a critical barrier and playing a central role in numerous pathologies [1]. Many diseases and lesions originate within the dermal layers, such as cancers and other tissue abnormalities [2,3]**.** These conditions disrupt the skin's structure and affect its mechanical properties, specifically its elasticity [4–7]. Consequently, depth-resolved elasticity estimation is essential for non-invasively monitoring disease progression and evaluating treatment effectiveness.

Elastography is an imaging modality that evaluates tissue mechanical properties by mapping spatial variations in stiffness [8–10]. Surface Acoustic Wave Optical Coherence Elastography (SAW-OCE) offers high spatial resolution for tracking wave propagation by Optical Coherence Tomography (OCT) [11–14]. In SAW-OCE, depth-resolved elasticity is characterized by converting the phase velocity of surface acoustic waves at specific tissue depths. In conventional implementations, phase velocity estimation involves a multi-step processing pipeline in which wave propagation is first captured in the spatial-temporal domain through phase differences between successive M-scans. The data are subsequently transformed into the frequency–wavenumber domain to extract phase velocity dispersion curves [15–18]. However, this approach is computationally redundant, and conventional methods require prior knowledge of material properties to map frequency to specific layers. Besides, it often yields inaccurate velocity estimates for both superficial and deeper regions because of the relatively low signal intensity. To address the loss of depth resolution inherent in conventional dispersion-based approaches, an alternative strategy is to directly estimate phase velocity from the spectral representation of depth-resolved complex signals. By analyzing the energy distribution in the frequency–wavenumber domain at specific depths, phase velocity can be recovered without relying on global dispersion curves, thereby enabling depth-resolved elasticity characterization.

Recently, deep learning–based approaches have been increasingly applied to optical coherence elastography (OCE) data processing, improving the efficiency of mechanical property estimation such as wave velocity [19] and tissue elasticity [20]. For example, Schlaefer’s group employed densely connected neural networks (DenseNets) [21] to analyze four-dimensional phase image volumes for estimating agar concentrations [22]. In subsequent work, they proposed three-dimensional spatial-temporal convolutional neural networks to estimate the elasticity of gelatin phantoms and porcine heart tissue. However, the reported mean absolute error (MAE) of 0.65 ± 0.81 m/s and relative MAE (RMAE) of 0.131 ± 0.162 m/s indicate only moderate predictive accuracy [20]. Zhang and Liao et al. developed the Velocity Prediction Network (VPNet) [19] and the Surface Acoustic Wave Prediction Network (SPNet) [23], which utilize raw phase data in the form of M–B scan sequences to estimate surface wave velocity. Although these models achieved promising results on phantom data, their performance were degraded when applied to *in vivo* human measurements, yielding a mean MSE of 0.172 and a mean MAE of 0.260. Transformer-based architectures have recently achieved remarkable success in computer vision [24], particularly through the Vision Transformer (ViT), which has demonstrated state-of-the-art performance in image classification [25], segmentation [26–28], and image-to-value regression tasks for stiffness estimation. In elastography, Mieling’s group further adapted ViT by using depth–time (A-line) sequences to estimate tissue elasticity, simplifying the scanning protocol while achieving competitive accuracy [29]. Nevertheless, this approach lacks depth-wise stiffness resolution and inherits the substantial computational complexity and hardware requirements of Transformer-based models, which limits its practicality for clinical applications.

To address these limitations, this study proposes an advanced hybrid model, termed Phase Velocity Network (PVNet), which integrates convolutional feature extraction with an efficient additive attention mechanism based on SwiftFormer [30]. By using phase images in the lateral–temporal domain as input to predict phase velocity at specific depths, the proposed approach improves estimation accuracy and robustness while preserving critical depth-resolved spatial information.

## 2. Method

### 2.1. Material

To validate the proposed depth-resolved phase velocity estimation framework, both tissue-mimicking phantoms and *in vivo* human skin measurements were conducted. Homogeneous and layered agar phantoms were used to quantitatively evaluate estimation accuracy and depth-wise sensitivity. Homogeneous agar phantoms (Fisher Scientific, no. A/1080/53) were fabricated with concentrations ranging from 1% to 5% at intervals of 0.5% (27 samples in total) and 1% TiO2 was added to enhance the optical scattering prior to gelation. For each concentration, samples were prepared using agar solutions from three independent batches, and each sample was measured three times to ensure repeatability. Two layered agar phantoms were additionally fabricated to evaluate phase velocity variations at material boundaries in Table 1.

**Table 1.** Composition of the layered agar phantom. Concentrations are expressed as agar weight per water volume (w/v %). Top: surface layer, Bottom: deeper layer

Amounts	Top (w/v)	Bottom (w/v)
2	2%	1%

For *in vivo* skin experiments, to assess clinical feasibility and robustness in complex biological tissues, twelve healthy young volunteers (aged 20–30 years) participated in this study under the approval of the Physical Sciences Ethics Committee (PSEC) of the University of York. All procedures conformed to the tenets of the Declaration of Helsinki. OCE measurements were performed at four anatomical locations for each subject: forearm, palm, back of the hand, and face. Each measurement was repeated three times at different positions to account for spatial variability. For both agar phantoms and human skin measurements, phase images were extracted uniformly along the depth direction. Specifically, starting from the sample or skin surface, 100 sequential depth-resolved phase images were acquired at equal depth intervals, where the signal-to-noise ratio (SNR) is relatively high, particularly in human skin. Then, surface flattening was applied to enable consistent depth-wise analysis across all experiments.

### 2.2. Experimental setup

#### 2.1.1. OCE system

**Fig. 1.**
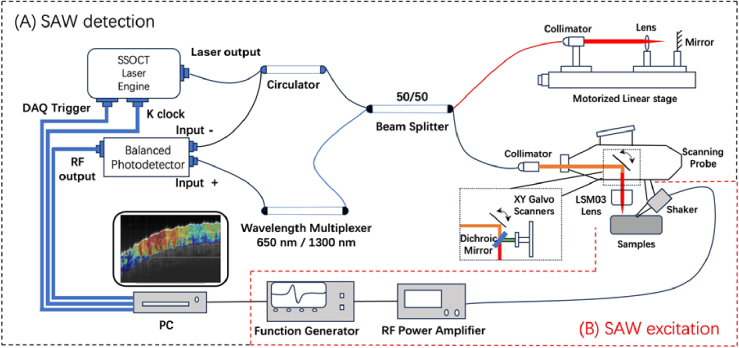
Schematic of the optical coherence elastography (OCE) experimental setup, consisting of (A) SAW detection based on swept-source OCT and (B) SAW excitation using a contact-based piezoelectric shaker.

The optical coherence elastography (OCE) experimental setup consisted of two main components in Fig. 1: swept-source optical coherence tomography (SS-OCT) detection and surface acoustic wave (SAW) excitation, as described previously [31]. The SS-OCT system operated at a sweep rate of 400 kHz, with a central wavelength of 1300 nm and a bandwidth of 100 nm. An M–B scanning protocol was employed to capture SAW propagation within a two-dimensional cross-sectional region of the sample. In this protocol, the laser scanning line defined the region of interest along the lateral direction of 5.1 mm with 8.6 µm lateral resolution, with a penetration depth of approximately 2 mm and an axial resolution of 4.7 µm in the sample. The resulting OCE volumetric dataset had dimensions of 384 × 600 × 600 (axial × lateral × time), which required 2 seconds per acquisition. For excitation part, the stimulation signal was a square wave at 4 kHz with an amplitude of 10 Vpp, which was applied to drive a mechanical shaker to generate surface acoustic waves (SAWs) propagating along the sample surface.

#### 2.1.2. Mechanical test system

The mechanical properties of the agar phantoms were characterized using a mechanical testing system (CellScale UniVert S2, CellScale, Canada) in compression mode. The agar samples used for mechanical testing were prepared from the same batch as those used in the OCE experiments to ensure consistency. Prior to each measurement, the load cell was zeroed to ensure accurate force recording. A ramp displacement control mode was applied with a total displacement of 1.5 mm at a compression rate of 0.5 mm/s. The Young’s modulus was determined from the slope of the linear region of the stress–strain curve, with a linear fitting coefficient (R^2^) greater than 0.9, using the CellScale Data Analysis Software and was subsequently converted to the corresponding phase velocity based on our group previous work [32].

### 2.3. Overview of phase velocity estimation framework

**Fig. 2.**
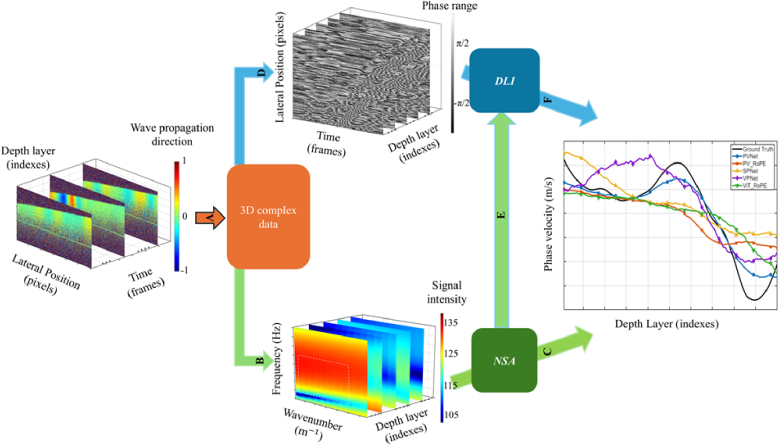
**Processing workflow: (A)** Acquisition of 3D complex OCE data showing SAW propagation. **(B)** Transformation to the frequency domain (white dash boxes are the ROI of frequency-wavenumber) via FFT after surface flattening etc. **(C)** Phase velocity estimation using NSA. **(D)** Extraction of phase images for DLI input. **(E)** Generation of training labels from NSA outputs. **(F)** Depth-resolved phase velocity estimated via DLI. The right panel summarizes the results of two baselines in terms of phase velocity and depth index, where DLI methods (PVNet, SPNet, VPNet, etc.) are quantitatively evaluated against the NSA baseline (ground truth).

Based on the theoretical analysis described in the Equation (S1-S7), the phase velocity estimation framework is divided into two complementary processes: Energy Distribution (ED)-based estimation and Angular Distribution (AD)-based estimation. The ED process estimates phase velocity by analysing the spectral energy concentration in the frequency domain, implemented as Numerical Spectral Analysis (NSA). In contrast, the AD process estimates phase velocity by exploiting the phase evolution of the propagating wave in the spatial-temporal domain, implemented as Deep Learning Inversion (DLI). An overview of the complete workflow is illustrated in Fig. 2. First, 3D complex Optical Coherence Elastography (OCE) data are acquired to track Surface Acoustic Wave (SAW) propagation where the colormap represents the propagation direction. To ensure accurate velocity estimation along the sample surface, the 3D volume is flattened based on surface detection (Fig. 2(A)). The complex data are then processed by algorithm described in Section 2.3.1 and transformed into the frequency domain via a Fast Fourier Transform (FFT) (Fig. 2(B)). Subsequently, NSA is performed to estimate the phase velocity at each depth (Fig. 2(C)). In a parallel branch, phase images are generated from the complex data to serve as inputs for the DLI model (Fig. 2(D)). The corresponding labels are derived from the NSA results (Fig. 2(E)) to facilitate velocity estimation via DLI (Fig. 2(F)). Finally, the depth-resolved phase velocity results are integrated, and the DLI performance is quantitatively evaluated against the NSA baseline.

#### 2.3.1. NSA implementation

The phase velocity at each depth layer is estimated using an NSA framework applied to the complex data after flattening sample surface. For each depth layer *z*, a local spatial–temporal region of interest (ROI) is extracted along the lateral ( x ) and temporal ( t ) dimensions. To improve robustness against noise and local fluctuations, spectral estimates are spatially averaged across adjacent depth slices within a radius of ±2 layers. Each slice is apodized using a separable Hann window in both spatial and temporal dimensions before transformation to minimize spectral leakage and then a two-dimensional Fourier transform (2D-FFT) is applied to each windowed slice. The magnitude spectra are accumulated and averaged across the selected depth window to form a stabilized spectral energy map S(k,f) with a predefined (k,f) region, where *k* denotes the wavenumber and *f* temporal frequency. In this study, the spectral coordinates (k,f) are determined using a weighted spectral centroid method. The central spectral energy is used to calculate the centroid coordinates and corresponding phase velocity:

(1) $$C_{p}=\frac{\frac{\sum f\cdot S(k,f)}{\sum S(k,f)}}{\frac{\sum k\cdot S(k,f)}{\sum S(k,f)}}$$

To obtain a stable and smooth depth-dependent phase velocity profile, robust locally weighted regression (rLOESS) with a window size of 30 samples is applied. This robust smoothing reduces the influence of residual outliers while preserving depth-wise trends. The resulting smoothed phase velocity profiles are treated as ground truth labels for subsequent deep learning training, with each profile paired with its corresponding phase map extracted from the original complex-valued data.

##### 2.3.1.1. Interface detection via piecewise linear regression

To quantitatively determine the boundary depth between the layers, we employed a piecewise linear regression (bilinear fitting function) on the depth-resolved phase velocity profile [33]. Assuming the mechanical properties within each layer are relatively consistent but distinct, the velocity distribution along the depth ( z ) can be modelled as two linear segments separated by a transition point ( $z_{break}$ ):

(2) $$v(z)=\left\{ \begin{array}{l} a_{1}z+b_{1}\;(z\leq z_{break})\\ a_{2}z+b_{2}\;(z>z_{break}) \end{array}\right.$$

where $a_{1}$ , $b_{1}$ and $a_{2}$ , $b_{2}$ are the linear coefficients for the upper and lower layers, respectively. This model allows for a discontinuity (velocity jump) at the interface, reflecting the abrupt change in tissue stiffness. The optimal interface depth was identified by minimizing the total Sum of Squared Errors (SSE) between fitting linear and phase velocity at each depth.

#### 2.3.2. DLI implementation

The deep learning inversion (DLI) framework was implemented using Python 3.13.9 with PyTorch 2.7.1 and CUDA 11.8. Model training was performed on a workstation equipped with two NVIDIA GeForce RTX 3090 GPUs. All models were trained using the mean squared error (MSE) loss function with the Adam optimizer. The implementation parameters were selected based on preliminary experiments for regression tasks. The Adam optimizer was chosen due to its fast convergence and robustness to gradient scaling. The initial learning rate was set to $1\times 10^{-4}$ , with a batch size of 128. Each training session was run for a maximum of 200 epochs, and early stopping was applied with a patience of 15 epochs based on validation loss to prevent overfitting.

##### 2.3.2.1. Training strategy and dataset composition

A two-stage training strategy was employed to improve model generalization. In the first stage, the model was pre-trained using a previously established dataset comprising both *in vivo* human data and tissue-mimicking phantoms. The dataset included multiple human anatomical locations, namely the face, back of the hand, palm, forearm, and acne-affected regions, as well as agar phantoms with concentrations ranging from 0.5% to 5% in increments of 0.5%. Randomly shuffled phase images were used as input to enhance robustness to spatial and temporal variability. This pre-training dataset consisted of 39,116 phase images for training and 4,889 phase images for validation while the training and validation splits were fixed and predefined. In the second stage, the model weights were further fine-tuned using a newly constructed mixed sample dataset (Material description in Section 2.1), which contained 20,319 training images. To avoid data leakage, the 5,079 validation images were exclusively used to evaluate model performance during training and to select optimal model checkpoints. To assess generalization capability, the trained models were evaluated on 4,077 testing phase images that were not used during training, including layered agar phantoms and *in vivo* human data from different anatomical locations. These test cases were used solely for performance evaluation and were excluded from both training and validation processes.

##### 2.3.2.2. Architecture of PVNet

**Fig. 3.**
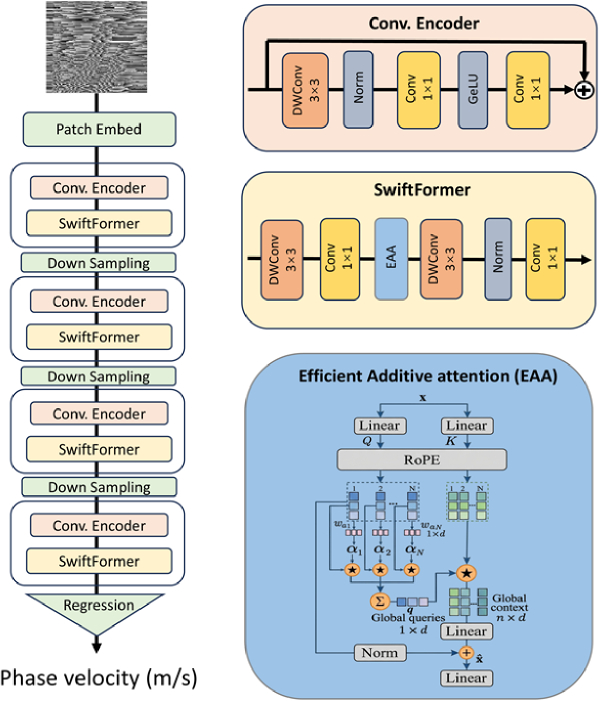
Overview of PVNet architecture (Left Column). The phase image (H: 320, W: 320, C: 1) at each depth as input to the patch embedding layer, and through four hierarchical encoding stages (Conv. Encoder and SwiftFormer) and the down sampling is used to reduce the half of size between the stages and a regression head to output the predicted phase velocity (m/s). The details of Conv. Encoder, SwiftFormer and EAA with RoPE are shown from top to bottom in sequence (Right Column).

We propose PVNet based on SwiftFormer [30], an efficient hybrid vision transformer architecture optimized for regression tasks. This model is applied to estimate the phase velocity relied on phase image at specific depth. The network adopts a hierarchical pyramid structure, integrating the local feature extraction capabilities of Convolutional Neural Networks (CNNs) [34] with the global context modeling of Transformers. To enhance the model’s sensitivity to spatial information, we incorporate Rotary Positional Embeddings (RoPE) [35] into the efficient additive attention mechanism. The overall architecture consists of a patch embedding stem, down sampling, four hierarchical encoding stages (Conv. Encoder and Swiftformer), and a regression head in Fig. 3. The detail of architecture is shown in Table S1 and S2. We adopted a hybrid CNN-Transformer architecture to simultaneously capture local phase details and global wave propagation contexts. Furthermore, the incorporation of RoPE enhances spatial sensitivity, ensuring precise velocity regression from position-dependent phase images.

(1) **ConvEncoder Block**
  The ConvEncoder serves as the primary local feature extraction module. It is designed to prioritize spatial locality and computational efficiency.
  Let the input feature map be $X\in R^{B\times C\times H\times W}$ . The ConvEncoder processes *X* through a sequence of depthwise and pointwise operations:
(3) $$X_{1}=Conv_{1\times 1}(BN(DWConv_{3\times 3}(X)))$$
(4) $$X_{out}=Conv_{1\times 1}(GELU(X_{1}))+X$$
  First, a depthwise convolution ( $DWConv_{3\times 3}$ ) is applied with kernel size ( 3×3 ), which acts as a spatial filter, this layer learns spatial patterns for each channel independently, significantly reducing parameter count compared to standard convolutions. This is followed by BatchNorm ( BN ) and a pointwise convolution ( $Conv_{1\times 1}$ ), which facilitates cross-channel information exchange. A GELU activation introduces non-linearity, and a final pointwise convolution projects the features to the desired output dimension. This structure effectively captures local texture and edge information of phase image.
(2) **SwiftFormer Encoder**
  The SwiftFormer Encoder Block is the fundamental architectural unit of the network. It begins with a combination of depthwise and pointwise layers to further refine spatial-channel dependencies. Critically, the feature map is then flattened into a sequence of tokens to be processed by the attention mechanism. The block integrates EAA augmented with RoPE to capture long-range dependencies. Finally, a Linear Block (consisting of point convolution, depthwise convolutiond and normalization) projects the features back to the original space. For an input tensor $X\in R^{B\times C\times H\times W}$ , the block processes features in the following sequence:
(5) $$X_{1}=Conv_{1\times 1}(DWConv_{3\times 3}(X))$$
(6) $$X_{attn}=EAA(RoPE(X_{1}))$$
(7) $$X_{out}=Conv_{1\times 1}(BN(DWConv_{3\times 3}(X_{attn})))$$
(3) **Efficient Additive Attention (EAA)**
  To resolve the computational bottleneck of standard Transformers, we employ Efficient Additive Attention. This module reduces the complexity from quadratic $O(N^{2})$ to linear O(N) with respect to the number of tokens. Let $Q,K\in R^{N\times D}$ be the Query and Key matrices after RoPE application. The attention process is defined as:
(8) $$\alpha =Softmax\left(\frac{Q\cdot w_{a}}{\sqrt{d}}\right)\epsilon \;R^{N\times 1}$$
(9) $$q=\sum_{i=1}^{N}(\alpha_{i}\cdot Q_{i})\;\epsilon \;R^{1\times D}$$
(10) Output=Linear(K⊙q)+Q
  First, the model computes the similarity between every *Q* token and $w_{a}$ to generate a saliency map ( α ). This map indicates which regions of the image are most important. Second, we compute a single Global Query Vector ( q ) by taking the weighted average of all *Q* tokens based on α . This vector summarizes the entire image context. Finally, this global context is broadcasted and multiplied element-wise with the *K*. This step modulates the local features using the global scene information, allowing the network to focus on relevant features for the regression task efficiently.
(4) **Rotary Positional Embedding (RoPE)**
  We incorporate Rotary Positional Embeddings (RoPE) to inject positional information into the network [35]. Unlike absolute positional encodings that are added to the input, RoPE encodes position by rotating the Q and K vectors in a geometric space. This rotation ensures that the interaction between tokens depends only on Q and K relative distances. Given a feature vector *x* and a spatial position index *m*, RoPE applies a rotation matrix. We group adjacent elements of *x* into pairs ( $x_{2i}$ , $x_{2i+1}$ ) and rotate them by an angle $\theta_{m}=m\cdot \theta_{i}$ :
(11) $$f_{RoPE}(x,m)=\left(\begin{array}{l} x_{2i}\\ x_{2i+1} \end{array}\right)\cdot \left(\begin{array}{cc} cos\;m\theta_{i} & -\sin m\theta_{i}\\ \sin m\theta_{i} & cos\;m\theta_{i} \end{array}\right)$$
  Where $\theta_{i}$ = $10000^{-2i/d}$

##### 2.3.2.3. Evaluation metrics

To evaluate the difference between the predicted phase velocity with ground truth, the Mean Absolute Error (MAE) function is employed, which provides a linear representation of the average error magnitude, offering a more robust and physically interpretable assessment of the model's accuracy.

## 3. Results

### 3.1. NSA results for homogeneous agar phantoms

**Fig. 4.**
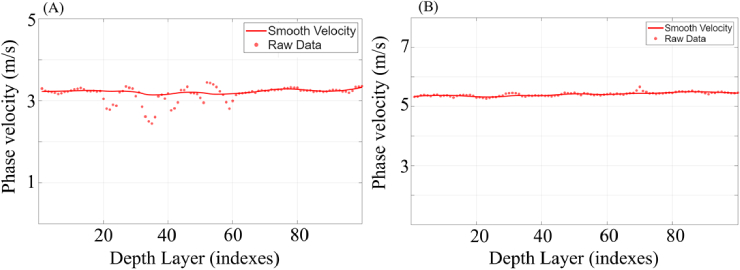
NSA-derived phase velocity profiles for homogeneous agar phantoms: (A) 1% agar and (B) 2% agar. Raw data represents the estimated phase velocity at each depth layer, while the smooth velocity curves are obtained using rLOESS smoothing. The horizontal axis indicates the depth layer index (1–100), where each interval corresponds to 4.7 µm, and the vertical axis denotes phase velocity (m/s).

The NSA-derived phase velocity profiles for homogeneous agar phantoms with 1% and 2% concentrations are shown in Fig. 4. For both agar samples, the phase velocity remains nearly constant along the depth direction, indicating uniform mechanical properties throughout the phantom. Specifically, the 1% agar phantom exhibited a phase velocity of approximately 3.2 m/s, while the 2% agar phantom showed a higher phase velocity of approximately 5.3 m/s across the analysed depth range. Based on NSA estimation results of these concentrations, the measured phase velocities were 3.8±0.81 m/s for the 1% agar and 6.46±0.41 m/s for the 2% agar. These NSA-derived values are in good agreement with independent mechanical tensile testing results, which were converted to equivalent phase velocities of 3.77±0.30 m/s and 6.60±0.56 m/s for the 1% and 2% agar phantoms, respectively.

### 3.2. NSA results for layered agar phantoms

**Fig. 5.**
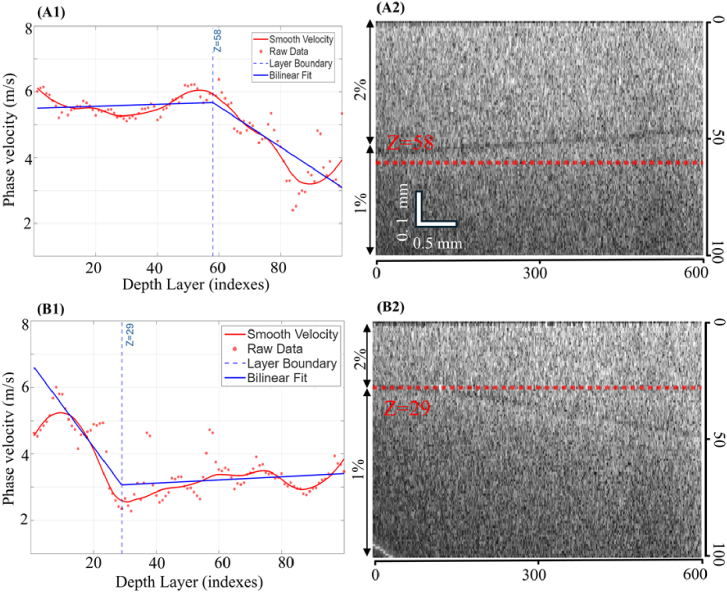
NSA results for two-layer agar phantoms (top layer: 2% agar; bottom layer: 1% agar) with different top-layer thicknesses. Phase velocity profiles as a function of depth are shown in (A1) and (A2) as a function of depth index (1–100), while the corresponding B-scan images are shown in (B1) and (B2). Red dashed lines indicate the detected interface locations based on bilinear fitting of the phase velocity profiles.

Layered agar phantoms were employed to evaluate the capability of NSA to detect abrupt mechanical transitions at material interfaces. Two-layer phantoms consisting of a 2% agar top layer and a 1% agar bottom layer were analysed in Table 1. For the first layered phantom with a relatively thick top layer, the phase velocity profile shown in Fig. 5(A1) exhibits an approximately constant velocity of ∼5.5 m/s in the upper region. A distinct transition occurs at a depth index of approximately 58, below which the phase velocity decreases to about 3.1 m/s, corresponding to the lower concentration agar layer. The corresponding two-dimensional B-scan image in Fig. 5(A2) shows a clear structural boundary, with the detected interface (red dashed line) closely matching the true physical interface. In the second layered phantom with a thinner top layer, the phase velocity profile in Fig. 5(B1) begins at approximately 5.2 m/s and transitions at a depth index of around 29, after which the velocity stabilizes near 3.0 m/s. The detected boundary location is again consistent with the interface observed in the corresponding B-scan image shown in Fig. 5(B2), where the red dashed line indicates the estimated interface position.

### 3.3. NSA results for different human skin positions

**Fig. 6.**
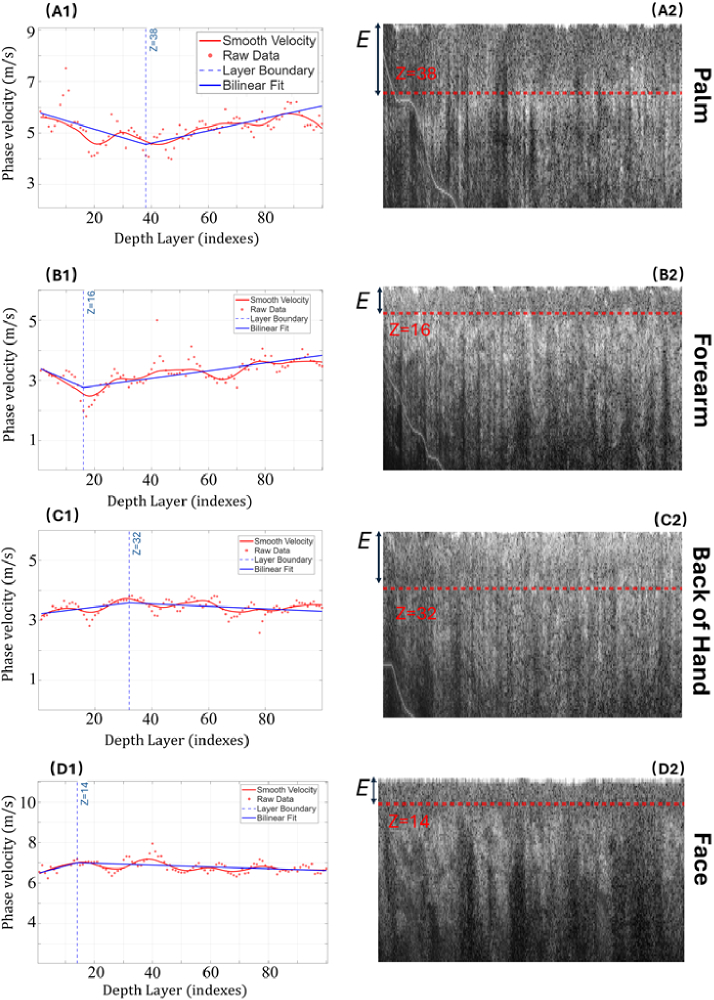
NSA-derived phase velocity profiles and corresponding B-scan images for different in vivo human skin positions: palm (A), forearm (B), back of the hand (C), and face (D). The phase velocity profiles and B-scan images are shown as a function of depth index (1–100). In the B-scan images, “E” denotes the epidermal layer, and red dashed lines indicate the detected interface determined by bilinear fitting.

NSA-based phase velocity analysis was performed on *in vivo* human skin at different anatomical locations, including the palm, forearm, back of the hand (BoH), and face. The depth-resolved phase velocity profiles for these positions are shown in Fig. 6. The average phase velocity values for the palm, forearm, back of the hand, and face are approximately 5 m/s, 3 m/s, 3.5 m/s, and 7 m/s, respectively. These values are consistent with previously reported measurements for corresponding skin regions. Although the phase velocity transitions in human skin are less abrupt than those observed in layered agar phantoms, the proposed NSA-based approach is still able to reliably identify interface locations across different tissue thicknesses.

For anatomical regions with thicker epidermal layers, such as the palm and back of the hand, the detected interface depths are located at approximately 35 depth indices, corresponding to a physical depth of about 0.165 mm. In contrast, for regions with thinner epidermal layers, including the forearm and face, the interface is detected at around 15 depth indices (approximately 0.071 mm). These results demonstrate the robustness of the bilinear fitting approach for interface detection in heterogeneous biological tissues.

### 3.4. DLI results for agar phantoms

The performance of the deep learning inversion (DLI) models was evaluated using phase images from agar phantoms with concentrations ranging from 1% to 5%, as well as layered agar phantoms in Table 2. The PV-RoPE, and PVNet were compared with network architectures, including VPNet [19], SPNet [23], ViT-RoPE [29] as per the published sources. Model performance was quantified using the MAE between predicted and reference phase velocities. As summarized in Table 2, the PVNet and PV-RoPE achieved the lowest MAE across all agar concentrations. Specifically, both models consistently achieved mean MAE values below 0.155 m/s for all homogeneous and layered agar samples.

**Table 2.** Mean absolute error (MAE, mean ± standard deviation, m/s) of different deep learning models for homogeneous and layered agar phantoms

	VPNet	SPNet	ViT-RoPE	PV-RoPE	PVNet
**Agar 1%**	0.16 ± 0.12	0.13 ± 0.11	0.15 ± 0.12	0.09 ± 0.09	**0.08** ± **0.07**
**Agar 2%**	0.21 ± 0.16	0.13 ± 0.11	0.14 ± 0.11	**0.08** ± **0.07**	0.09 ± 0.07
**Agar 3%**	0.30 ± 0.23	0.21 ± 0.18	0.30 ± 0.33	**0.13** ± **0.13**	0.16 ± 0.17
**Agar 4%**	0.34 ± 0.28	0.22 ± 0.20	0.42 ± 0.52	**0.15** ± **0.16**	0.18 ± 0.22
**Agar 5%**	0.29 ± 0.22	0.26 ± 0.22	0.19 ± 0.19	**0.13** ± **0.14**	0.14 ± 0.17
**Layered Agar**	0.29 ± 0.25	0.19 ± 0.16	0.35 ± 0.31	**0.14** ± **0.15**	0.15 ± 0.18

^a^Bold values mean the lowest MAE

PVNet demonstrated superior performance for lower-concentration agars (1% and 1.5%), while PV-RoPE achieved the best performance for the remaining concentrations. In contrast, VPNet exhibited substantially higher errors, particularly for higher agar concentrations (>2%), where the MAE exceeded 0.250 m/s.

### 3.5. DLI results for in vivo human skin

The trained DLI models were further evaluated using *in vivo* human skin data from different anatomical locations. The quantitative MAE results for each model and skin position are summarized in Table 3. Among all evaluated models, PVNet achieved the best overall performance, with MAE values below 0.1 m/s for all skin locations except the palm, where a higher error of 0.32±0.42 m/s was observed. Notably, other models also exhibited degraded performance at the palm location, with MAE values of approximately 0.55 m/s, suggesting increased structural complexity and variability in this region. VPNet exhibited the poorest performance overall across all skin positions with mean MAE 0.29 ± 0.27 m/s.

**Table 3.** Mean absolute error (MAE, mean ± standard deviation, m/s) of different deep learning models for in vivo human skin at different anatomical locations

	VPNet	SPNet	ViT-RoPE	PV-RoPE	PVNet
**Forearm**	0.18 ± 0.16	0.15 ± 0.14	0.18 ± 0.16	0.12 ± 0.11	**0.09** ± **0.09**
**Palm**	0.59 ± 0.60	0.57 ± 0.60	0.56 ± 0.62	0.46 ± 0.56	**0.32** ± **0.42**
**BoH**	0.14 ± 0.12	0.12 ± 0.11	0.13 ± 0.12	0.10 ± 0.08	**0.08** ± **0.07**
**Face**	0.23 ± 0.18	0.16 ± 0.13	0.14 ± 0.12	0.10 ± 0.10	**0.09** ± **0.09**

^a^Bold values mean the lowest MAE

### 3.6. DLI results for layered agar phantoms

**Fig. 7.**
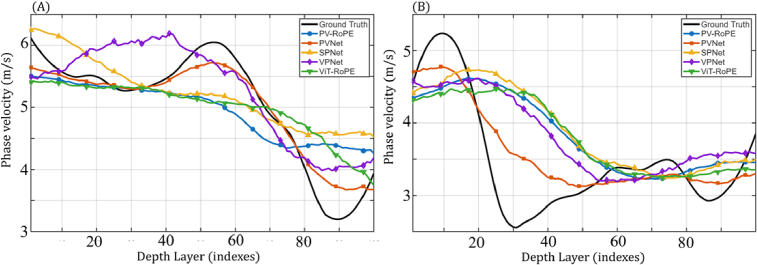
Depth-resolved phase velocity estimation for layered agar phantoms (top layer: 2% agar; bottom layer: 1% agar) with (A) thicker and (B) thinner top-layer thicknesses. NSA-derived phase velocity profiles are used as ground truth. Predicted results from PVNet, PV-RoPE, SPNet, VPNet, and ViT-RoPE are shown for comparison.

To evaluate the performance of the deep learning inversion (DLI) models on layered media, quantitative comparisons were conducted between the DLI-predicted phase velocity profiles and the NSA-derived results, which were treated as ground truth. Additional layered agar phantoms consisting of a 2% agar top layer and a 1% agar bottom layer were tested with different top-layer thicknesses. The Fig. 7(A) and Fig. 7(B) show the depth-resolved phase velocity estimates produced by different models for layered agar phantoms with thicker and thinner top layers, respectively. For both configurations, PVNet demonstrates the closest agreement with the ground truth, accurately following the phase velocity variation trend across depth.

Quantitative performance metrics, including mean absolute error (MAE), root mean square error (RMSE), are summarized in Table 4. For the layered agar with a thicker top layer (A), PVNet achieves the lowest MAE (0.17 ± 0.15 m/s) and RMSE (0.23 m/s), substantially outperforming all other models. In contrast, PV-RoPE exhibits noticeably higher errors, with MAE values of 0.48 ± 0.38 m/s and 0.60 ± 0.54 m/s for agar (A) and (B), respectively. For the layered agar with a thinner top layer (B), model performance generally degrades across all architectures, highlighting the increased difficulty of phase velocity estimation in thin-layer configurations. In this case, all models except PVNet showing poor agreement with the ground truth. PVNet remains robust, achieving a relatively low RMSE of 0.40 m/s, while other models, including PVNet-RoPE, failed to reconstruct the depth-dependent velocity profile.

**Table 4.** Performance comparison of different models for layered agar phantoms shown in Fig. 7. Metrics include MAE (mean ± standard deviation, m/s), RMSE (m/s)

	Agar (A)		Agar (B)	
Model	MAE	RMSE	MAE	RMSE
**SPNet**	0.53 ± 0.39	0.66	0.63 ± 0.58	0.86
**VPNet**	0.50 ± 0.34	0.61	0.58 ± 0.52	0.78
**PVNet**	**0.17** ± **0.15**	**0**.**23**	**0.28** ± **0.29**	**0**.**40**
**PV-RoPE**	0.48 ± 0.38	0.61	0.60 ± 0.54	0.80
**ViT-RoPE**	0.45 ± 0.37	0.59	0.61 ± 0.58	0.84

^a^Bold values indicate the lowest MAE and RMSE

### 3.7. Baseline comparison

Table 5 summarizes the computational complexity, inference efficiency of the evaluated models and accuracy (includes the MAE of agar and human sites) of the evaluated models under a batch size of 128 and an input resolution of 320 × 320 pixels. The proposed NSA achieves an inference speed of 13.79 FPS, it incurs significantly higher computational costs than PVNet and the other benchmark models. PVNet employs an additive attention backbone, resulting in a higher number of parameters than CNN-based models such as VPNet and SPNet, while maintaining moderate computational complexity (1.51 GFLOPs). Compared with VPNet, PVNet shows lower inference speed but achieves a balanced trade-off between accuracy and efficiency. PV-RoPE has a similar number of parameters and GFLOPs as PVNet but exhibits reduced FPS due to additional positional encoding overhead. Scaled variants of PVNet (PVNet-S and PVNet-L) demonstrate that the model can be adapted to different computational constraints by adjusting model size and the architecture of them are shown in Table S1 and S2.

**Table 5.** Comparison of model complexity, inference efficiency and accuracy for phase image regression

Model	Parameters (M)	FLOPs (G)	FPS	MAE (m/s)
**VPNet**	1.94	0.17	26594.4	0.277 ± 0.103
**SPNet**	1.5	0.25	4688.4	0.202 ± 0.133
**ViT-RoPE**	3.23	2.58	2155.5	0.252 ± 0.134
**PVNet**	165.66	1.51	2084	**0.134** ± **0.093**
**PVNet-S** ^*a*^	41.43	0.39	3648.4	0.162 ± 0.105
**PVNet-L** ^*a*^	167.08	2.5	1087.6	0.145 ± 0.095
**PV-RoPE**	165.66	1.51	1681.4	0.140 ± 0.111

^*a*^ S and L denote small and large variants of PV-net, respectively

^b^Bold values indicate the lowest MAE

## 4. Discussion

The phase velocity estimation results consistently demonstrate that the proposed NSA framework enables accurate and depth-resolved across both homogeneous and layered media. In homogeneous agar phantoms, the phase velocities derived from the NSA method show close agreement with those obtained from mechanical testing. Compared with traditional phase velocity estimation methods in the frequency domain, the proposed NSA method offers two main advantages [15–18]. First, the proposed method directly estimates phase velocity at each minimal depth-resolved pixel from the spectrum of the acquired 3D complex data. In contrast, conventional methods require multiple post-processing steps, from spatial to frequency and back to spatial domains, and then estimate depth-dependent phase velocity based on known physical parameters of the sample [36]. Second, NSA enables reliable phase velocity estimation in both superficial and deeper layers by improving signal-to-noise ratio (SNR) of depth layers through layered averaging and weighted centroid strategies, whereas traditional methods exhibit reduced accuracy and robustness in these regions due to low SNR. For the NSA strategy, the Fourier transform redistributes the signal energy into discrete frequency components, allowing coherent wave motion to be separated from broadband noise. In the time domain, signal is imposed directly by noise in the displacement waveform, which makes phase estimation sensitive to random fluctuations. Additionally, the phase velocity estimation is less sensitive to amplitude variations. Even when signal amplitude attenuates with depth, the phase information at the excitation frequency can remain stable, allowing reliable velocity estimation under moderate SNR conditions.

Importantly, the layered agar experiments further highlight the depth sensitivity of NSA. The clear breakpoint observed at the interface between agar layers confirms that the method can resolve mechanical discontinuities along the depth direction. Such capability is particularly relevant for biological tissues, where stiffness often varies with depth. Nevertheless, conventional phase velocity estimation relies on a smoothed frequency dependent dispersion curve, and therefore fails to capture depth-specific mechanical information at material or tissue interfaces.

While NSA establishes a robust physic-based benchmark for depth-resolved elastography, its practical application is partially constrained by the requirement for manual frequency range selection. The proposed DLI framework complements NSA by automating this inversion process, estimating directly from the time-space complex data. The results of PVNet consistently outperformed implemented models based OCE models across a wide range of agar concentrations and in human *in vivo* skin data. The improved performance is primarily attributed to the adoption of the efficient additive attention (EAA) mechanism, which increases the effective learning capacity by introducing additional training parameters for global feature aggregation. Compared with conventional attention designs (SPNet and ViT-RoPE), EAA enables more expressive modeling of phase details. While rotary positional embedding is incorporated to preserve positional consistency, it does not constitute the main source of performance improvement.

However, the performance of the proposed NSA method is fundamentally constrained by the weighted centroid estimation strategy, which is highly sensitive to the selection of frequency and wavenumber search ranges. As a result, the definition of appropriate spectral input windows constitutes a critical prerequisite for robust and stable phase velocity estimation. Since mechanical testing cannot be performed in *in vivo* human skin experiments, the reliability of phase velocity estimation was inferred from reference recordings [15]. Accordingly, agar phantoms with controlled and varying concentrations were used as a prerequisite to validate the proposed methodology before its application to *in vivo* measurements. In addition, the NSA method did not consistently resolve distinct depth-dependent phase velocity variations in the skin measurements. This limitation is likely due to the spatial averaging inherent in the spectral estimation across adjacent depth slices, together with the relatively small thickness of the epidermal layer compared to the SAWs wavelength. The use of chirped excitation signals represents a potential method for improvement [37,38], as it may enhance the signal-to-noise ratio (SNR) across depth and frequency, thereby facilitating more reliable velocity estimation. With respect to the deep learning inversion (DLI) framework, the proposed PVNet architecture demonstrated superior performance compared with existing models across multiple experimental settings. Nevertheless, accurate inversion in the presence of extremely thin superficial layers remains challenging, this limitation may arise from the coexistence of guided wave modes, rather than purely surface acoustic waves (SAWs), which complicates the phase velocity interpretation [39]. Moreover, despite its improved estimation accuracy, PVNet exhibits lower computational efficiency than conventional CNN-based models, implying a greater reliance on high-performance computing resources for practical deployment.

## 5. Conclusion

This work establishes a workflow for OCE analysis combining Numerical Spectral Analysis (NSA) and Deep Learning Inversion (DLI). Unlike traditional methods relying on complex dispersion curve calculations, our NSA approach directly visualizes depth-resolved phase velocity, effectively delineating boundaries in layered agar and human skin. These findings suggest that the proposed NSA and DLI (PVNet) pipeline offers a robust and computationally efficient solution for depth-resolved stiffness characterization in OCE, with strong potential for clinical applications requiring reliable detection of layered tissue structures. This work enables clinically relevant depth-resolved phase velocity estimation in layered biological tissues, including human skin, using an efficient additive attention network integrated with SAW-OCE, supporting accurate stiffness assessment for disease-related structural alterations across tissue layers.

## Supplemental information

Supplement 1 Theory basis of phase velocity estimation and PVNet construction https://doi.org/10.6084/m9.figshare.31744231

## Data Availability

Data underlying the results presented in this paper are not publicly available at this time but may be obtained from the authors upon reasonable request.

## Supplemental document

See Supplement 1 for supporting content.
