# Supplementary material for: Depth-resolved phase velocity estimation in layered tissue based on an efficient additive attention network with surface acoustic wave – optical coherence elastography
Source: Biomed Opt Express. 2026 Apr 21;17(5):2533–48. doi: 10.1364/BOE.593027 (PMC13178624; doi:10.1364/BOE.593027)
Supplement: Supplement 1 [file boe-17-5-2533-s001.pdf]

Supplemental Document

Biomedical Optics EXPRESS

Depth-resolved phase velocity estimation in layered tissue based on an efficient additive attention network with surface acoustic wave – optical coherence elastography: supplement

GUANGYU ZHANG,[1] JINPENG LIAO,[1,2] ZHENGSHUYI FENG,[1] KATRIEN VAN BOCXLAER,[3] ALISON M. LAYTON,[3] CHUNHUI LI,[2,*] AND ZHIHONG HUANG[1]

[1] *Healthcare Engineering, School of Physics and Engineering Technology, University of York, UK*
[2] *Biomedical Engineering, School of Science and Engineering, University of Dundee, UK*
[3] *Hull York Medical School, University of York, York, UK*
[*] *c.li@dundee.ac.uk*

This supplement published with Optica Publishing Group on 21 April 2026 by The Authors under the terms of the Creative Commons Attribution 4.0 License in the format provided by the authors and unedited. Further distribution of this work must maintain attribution to the author(s) and the published article's title, journal citation, and DOI.

Supplement DOI: https://doi.org/10.6084/m9.figshare.31744231

Parent Article DOI: https://doi.org/10.1364/BOE.593027

1. Theory

1.1 Theory basis of phase velocity estimation

In this study, phase velocity estimation is performed through two complementary pipelines. The first pipeline follows a physics-based spectral analysis framework, in which depth-resolved phase velocity is directly extracted from the energy distribution (ED) in the frequency–wavenumber (f,k) domain. The second pipeline adopts a data driven strategy, where phase velocity is estimated from depth-resolved phase distribution (AD) using a deep learning network.

1.1.1 Based on Energy Distribution (ED)

The complex-valued signal is modeled as an analytic representation of a harmonic wave propagating in the spatial-temporal domain (x,t). By applying a two-dimensional fast Fourier transform (2D FFT), the signal is transformed from the (x,t) domain into the angular wavenumber–angular frequency domain (K,ω). In this domain, the signal energy is concentrated around a specific spectral coordinate corresponding to the angular wavenumber k_{rad} and angular frequency ω of the propagating wave.

The complex signal can be expressed as [32]:

$$s(x,t) = Ae^{i(k_{rad}x-\omega t+\phi_0)} \text{(S1)}$$

where A is the amplitude, k_{rad} is the angular wavenumber (rad/m), ω is the angular frequency (rad/s), and ϕ_0 is the initial phase.

After applying the 2D FFT:

$$\mathcal{F}\{s(x,t)\} = Ae^{i\phi_0}\delta(K-k_{rad}, W-\omega) \text{(S2)}$$

where $\delta(\cdot)$ denotes the Dirac delta function.

Taking the magnitude yields:

$$|\mathcal{F}\{s(x,t)\}| = A \cdot \delta(K-k_{rad}, W-\omega) \text{(S3)}$$

which removes the signal magnitude while retaining the correct spectral location. The phase velocity is then calculated as [33]:

$$C_p = \frac{\omega}{k_{rad}} = \frac{2\pi f}{2\pi k} = \frac{f}{k} \text{(S4)}$$

where f is the frequency and k is the wavenumber.

1.1.2 Based on Angular Distribution (AD)

In the angular distribution approach, phase velocity estimation is performed by directly analyzing the phase evolution of the complex signal. Applying the angle operator to the complex signal gives:

$$\Phi(x,t) = \angle(Ae^{i(k_{\text{rad}}x-\omega t+\phi_0)}) = k_{\text{rad}}x - \omega t + \phi_0 \text{(S5)}$$

Rearranging the equation:

$$x = \frac{\Phi(x,t)-\phi_0}{k_{\text{rad}}} + \frac{\omega}{k_{\text{rad}}} \text{(S6)}$$

from which the phase velocity is obtained as:

$$C_p = \frac{\omega}{k_{\text{rad}}} = \frac{2\pi f}{2\pi k} = \frac{f}{k} \text{(S7)}$$

Although the ED process removes the signal magnitude and the AD process discards the initial phase information, both methods preserve the essential propagation parameters k_{rad} and ω. In this section, we establish the theoretical equivalence between ED and AD representations for phase velocity

estimation. This analysis provides a unified physical foundation that bridges the spectral analysis module and the proposed learning-based inversion network.

Table S1. Architectural details of PVNet variants for ablation study

Stage	Input Resolution	Operation / Module	PVNet-S(C=[16,32,64,128])	PVNet (Base) (C=[32,64,128,256])	PVNet-L (C=[32,64,128,256])
Input	320×320	Grayscale Image	1	1	1
Patch Embedding	320×320	Conv 4×4, stride 4 + DWConv	C1 = 16 → 16	C1 = 32 → 32	C1 = 32 → 32
Stage 1	80×80	SwiftFormer Blocks ×2	16 → 16	32 → 32	32 → 32
Downsample 1	80×80	Conv 3×3, stride 2	16 → 32	32 → 64	32 → 64
Stage 2	40×40	SwiftFormer Blocks ×2	32 → 32	64 → 64	64 → 64
Downsample 2	40×40	Conv 3×3, stride 2	32 → 64	64 → 128	64 → 128
Stage 3	20×20	SwiftFormer Blocks ×2	64 → 64	128 → 128	128 → 128
Downsample 3	20×20	Conv 3×3, stride 2	64 → 128	128 → 256	128 → 256
Stage 4	10×10	SwiftFormer Blocks ×2	128 → 128	256 → 256	256 → 256
Regression Head	10×10	Global AvgPool + FC	128 → 1	256 → 1	256 → 1

*Block = Conv Encoder + Efficient Additive Attention

*FC = Fully Connected Layer

Table S2. Architectural Configuration of PVNet Variants

Model	Blocks per Stage	Channel Dimensions	Attention Heads
PVNet-S	[2, 2, 2, 2]	[16, 32, 64, 128]	4
PVNet (Base)	[2, 2, 2, 2]	[32, 64, 128, 256]	4
PVNet-L	[4, 4, 4, 4]	[32, 64, 128, 256]	4
